# Enforcement may crowd out voluntary support for COVID-19 policies, especially where trust in government is weak and in a liberal society

**DOI:** 10.1073/pnas.2016385118

**Published:** 2020-12-21

**Authors:** Katrin Schmelz

**Affiliations:** ^a^Department of Economics, University of Konstanz, D-78457 Konstanz, Germany;; ^b^Thurgau Institute of Economics, CH-8280, Kreuzlingen, Switzerland

**Keywords:** social norms, institutions, state capacities, cooperation, crowding out intrinsic motivation

## Abstract

Effective states govern by some combination of enforcement and voluntary compliance. To contain the COVID-19 pandemic, a critical decision is the extent to which policy makers rely on voluntary as opposed to enforced compliance, and nations vary along this dimension. While enforcement may secure higher compliance, there is experimental and other evidence that it may also crowd out voluntary motivation. How does enforcement affect citizens’ support for anti–COVID-19 policies? A survey conducted with 4,799 respondents toward the end of the first lockdown in Germany suggests that a substantial share of the population will support measures more under voluntary than under enforced implementation. Negative responses to enforcement—termed control aversion—vary across the nature of the policy intervention (e.g., they are rare for masks and frequent for vaccination and a cell-phone tracing app). Control aversion is less common among those with greater trust in the government and the information it provides, and among those who were brought up under the coercive regime of East Germany. Taking account of the likely effectiveness of enforcement and the extent to which near-universal compliance is crucial, the differing degrees of opposition to enforcement across policies suggest that for some anti–COVID-19 policies an enforced mandate would be unwise, while for others it would be essential. Similar reasoning may also be relevant for policies to address future pandemics and other societal challenges like climate change.

## Significance

This paper makes three contributions. First, it provides insights from Germany on people’s agreement with policy choices that all countries face in addressing the COVID-19 pandemic. My findings point to dimensions relevant for policy makers when deciding between voluntary as opposed to enforced measures. These insights include the essential role of trust in government. Second, the paper contributes to the small but important literature on the intersection of policy design, state capacities, and the interplay of obedience and voluntary compliance. Third, my finding that even 30 y after reunification those who have experienced state coercion in East Germany are less control-averse concerning anti–COVID-19 measures than West Germans contributes to the literature on endogenous preferences and comparative cultural studies.

What is the appropriate role of enforcement and explicit incentives as opposed to people’s sense of responsibility and voluntary compliance in combating the COVID-19 pandemic? Strongly enforced mobility restrictions have been imposed in many countries and have been shown to be highly effective in controlling the spread of COVID-19 in China (1). In a similar vein, some economists have proposed systems to incentivize desired citizen behavior during the COVID-19 crisis (2, 3).

However, we also observe substantial voluntary cooperation in the absence of enforcement or material incentives. For example, people in the United States started staying at home well before stay-at-home orders were issued and this behavior did not increase with the enforcement of lockdowns (4). Also, Germany succeeded in containing the first wave of the pandemic with a rather mild lockdown compared to its European neighbors. Less-stringent lockdowns in democracies have been found to be at least as effective in reducing movement as more-stringent lockdowns in autocracies (5). Reunified Germany provides a valuable lens to study the impact of recent differences in political regimes on the cultural environment in which anti–COVID-19 policies are implemented.

Most anti–COVID-19 policies share the fundamental structure of public goods dilemmas where all-encompassing cooperation maximizes the well-being of all citizens, but since cooperation is costly each individual has an incentive to free ride on others’ cooperation. Experiments with public-goods games around the world have shown that in the absence of punishment of free riding substantial levels of initial cooperation typically decline as contributors become discouraged or angered by those not contributing (6). According to a large literature on cooperation and punishment, people expect enforcement to ensure higher cooperation in the population. For example, the belief that most others will cooperate encourages conditional cooperators to do the same (7–9). This suggests that average agreement to follow anti–COVID-19 measures should be higher if a measure is enforced than if it remains voluntary.

On the other hand, enforcement and incentives can reduce intrinsic motivation, a phenomenon termed “motivational crowding out.” Evidence was provided by psychologists decades ago under the umbrella of “self-determination theory,” distinguishing between autonomous and controlled motivation (10, 11). More recently, this phenomenon has also been emphasized by political scientists (12) and found in behavioral experiments by economists (13, 14). The possibility that the effectiveness of an enforcement-based approach might be compromised because it crowded out voluntary commitment has also been termed “control aversion” (15). There is evidence that the frequency of control-averse types varies across populations (16, 17) and that control aversion can be identified in neuropsychological correlates (18).

This paper explores the relative importance of these two countervailing effects of enforcement on motivation with respect to measures combating COVID-19 in five domains: contact-tracing apps, vaccination, contact restrictions, limitations on travel, and wearing masks. In a Germany-wide online survey with 4,799 respondents (*SI Appendix*, Table S1), I investigate the extent to which people agree to follow those measures under two conditions: if the regulation is strongly advised by the government but remains voluntary and if it is enforced.

## The Survey and Results

To study the possibility that enforcement may crowd out civic values it is essential not to confound social motives for adopting a measure on the one hand with obedience to the law on the other. Therefore, my questions ask about the respondent’s attitude toward the measure and not whether a person would comply with a legally imposed and enforced measure. Details about the rationales behind the survey questions are provided in *Materials and Methods*.

My survey question on the contact-tracing app, for example, reads as follows: “We are currently discussing an app that accesses the movement and contact data of mobile phones to inform users anonymously about a possible infection. This app is more useful the more people use it. To what extent do you agree to use this app yourself if: … using the app is strongly recommended by the government but remains voluntary? … using the app is compulsory and checked by the government?” Answers were given on a 5-point Likert scale ranging from 0 (“not agree at all”) to 4 (“fully agree”). The questions on the other four domains follow the same scheme (*SI Appendix*, *Survey questions*).

A series of control variables including sociodemographic information and trust were also elicited in the survey. Participation was incentivized with tokens which could be exchanged for goods. The survey was conducted at a time when stay-at-home orders were in place, national borders were closed, the wearing of masks had been compulsory for a few days, vaccine development was in progress, and contact-tracing apps were being discussed (*SI Appendix*, *Timeline* and Fig. S1).

**Fig. 1.**
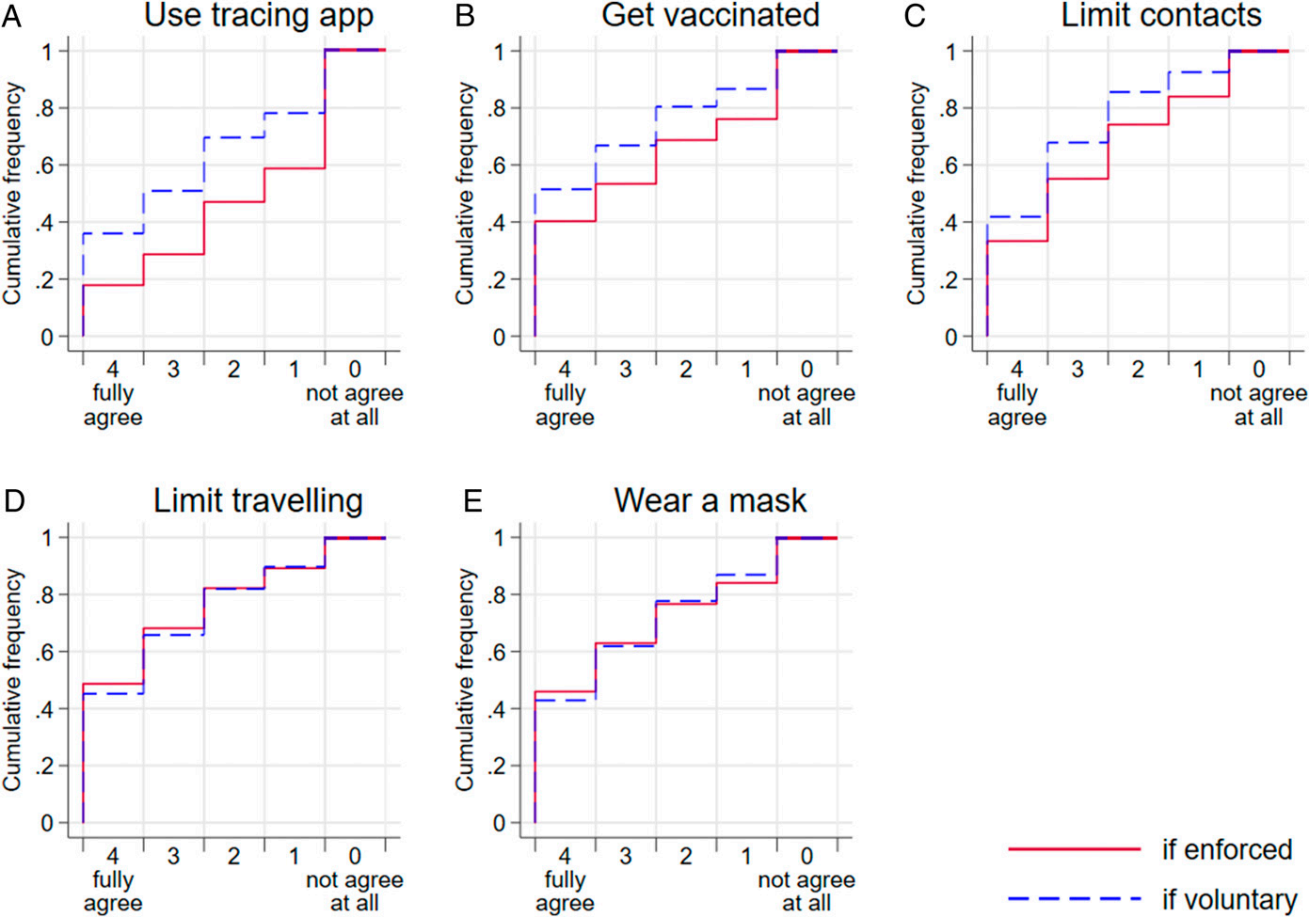
Cumulative distributions of agreement under the enforced vs. voluntary implementation of the five measures (panels *A–E*). For example, *A* shows that 18% and 36%, respectively, of respondents fully agree to use a contact-tracing app if it is enforced and voluntary, respectively. The sum of those expressing either agreement level 3 or 4 amounts to 29% and 51%, respectively, in case of enforcement and voluntariness, respectively. Strongest opposition (level 0) was expressed by 41% and 22%, respectively, under enforced and voluntary implementation, respectively (the final step in the upper left graph). The sample sizes are as follows. Use tracing app: *n* = 4,787 (if voluntary) and *n* = 4,777 (if enforced); Get vaccinated: *n* = 4,787 (if voluntary) and *n* = 4,786 (if enforced); Limit contacts: *n* = 4,790 (if voluntary) and *n* = 4,792 (if enforced); Limit traveling: *n* = 4,784 (if voluntary) and *n* = 4,794 (if enforced); Wear a mask: *n* = 4,776 (if voluntary) and *n* = 4,781 (if enforced).

The corona warning app was launched on a voluntary basis in Germany on 16 June, more than a month after the survey was completed. Within the first 6 wk it had been installed on 16.4 million smartphones (19). The number of active and compatible smartphones in Germany endowed with the required low-energy Bluetooth function is estimated by the leading newspaper *Der Spiegel* to be between 44 and 45 million (20). Based on these estimates, the number of downloads corresponds to 37% of those who could install the app, which is virtually identical with the share of 36% of my survey respondents who fully agree to use the app in case it remains voluntary (Fig. 1). I address further aspects of survey validity in *SI Appendix*, *Validity of the Survey Results*.

## Average Agreement Is Never Higher under Enforcement

As shown in the cumulative distributions of Fig. 1, I never observe substantially higher agreement under control. Voluntary agreement is always either higher than agreement under enforced measures (contact-tracing app, limiting contacts, and vaccination; Fig. 1 *A*–*C*) or virtually identical (limiting travels and wearing masks; Fig. 1, *D* and *E*).

Across all domains, roughly 50% to 70% of respondents strongly agree to adhere to voluntary anti–COVID-19 regulations (Fig. 1, blue lines, levels 3 and 4). The drop in agreement under control is most striking for the contact-tracing app where less than 20% totally agree under enforcement, while enforcement provokes strongest opposition (level 0) among more than 40% of the sample. This observation is crucial for policy makers as the success of such an app essentially requires its use by a substantial share of the population (21).

## Control Aversion, Especially for Contact-Tracing App

**Fig. 2.**
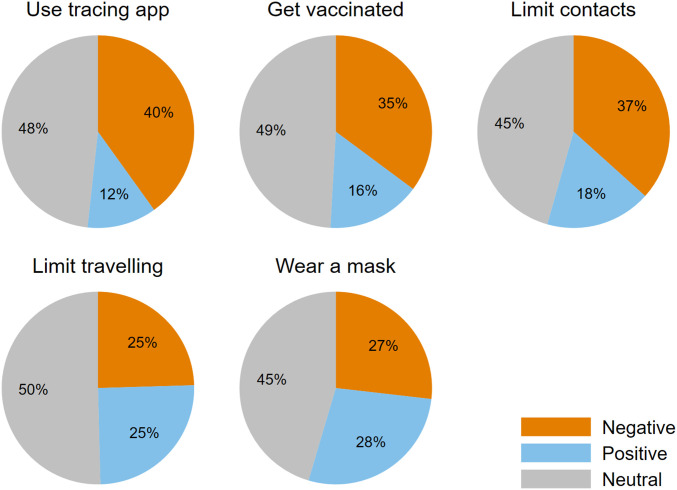
Shares of types of responses to enforced vs. voluntary policies. Responses to enforcement are negative (neutral and positive, respectively) if agreement is lower (equal to and higher, respectively) in case of enforced compared to voluntary implementations of a measure. Negative responses are termed control aversion. The sample sizes are as follows. Use tracing app: *n* = 4,770; Get vaccinated: *n* = 4,776; Limit contacts: *n* = 4,783; Limit traveling: *n* = 4,781; Wear a mask: *n* = 4,769. The underlying choice distributions are provided in *SI Appendix*, Fig. S3.

Fig. 2 shows that control aversion, that is, lower agreement under enforced than under voluntary conditions, occurs across all policies (orange slices), with its frequency varying between 25% (traveling) and 40% (app). Depending on the measure, relatively few—between 12% (app) and 28% (masks)—respond positively to control, that is, their agreement is greater under compulsory instead of voluntary implementations (blue slices). Roughly half of the sample is neutral with respect to (non)enforcement, expressing the same level of agreement to follow a measure irrespective of whether it is implemented voluntarily or compulsory (gray slices). Negative responses (control aversion) are more frequent than positive responses to control for the app, vaccination, and contact limitations, while positive and negative responses to control cancel out each other for travel restrictions and masks. Thus, average agreement is either higher under voluntary regulations (app, vaccination, and contact limitations) or similar if enforced and if voluntary (travel limitations and masks), as shown in *SI Appendix*, Fig. S2. This observation is corroborated statistically by two-sided sign tests, revealing that the difference between agreement in case of voluntary and enforced implementation is highly significant for contact-tracing apps, vaccination, and contact limitations (*P* < 0.0001) but insignificant for travel restrictions (*P* = 0.622) and masks (*P* = 0.389).

## Consistency of Responses to Enforcement across Policies

To assess the consistency with which individuals respond negatively, neutrally, and positively to enforcement, I count the number of domains in which a participant provides such a response (*SI Appendix*, Fig. S4). Control aversion can be associated with a relatively persistent type: 7% respond negatively to enforcement on all five measures and 29% are control-averse in the majority of domains (i.e., at least three out of five). The type of respondents expressing the same level of agreement independent of the implementation strategy is also fairly robust: 15% always respond neutrally to enforcement and 47% do so for the majority of measures. Positive responses are more rare. Hardly any participant (2%) expresses higher agreement under enforcement than under voluntary implementations across all measures, and only 15% do so in at least three domains.

A principal component analysis on the differences between voluntary agreement and agreement in case of enforcement shows substantial and similar weights across all five domains on the first component, accounting for 52% of the variance. The second component explains 18% of the variance and indicates that the domains tracing app and vaccination are different from the other three domains (*SI Appendix*, Fig. S5). These correlated negative responses might be directed toward the privacy-intrusive nature of enforcement in these cases, though the domains also differ on some other dimensions (*SI Appendix*, Table S2).

## Trust in Government, Communist Experience, and Health Issues Reduce Control Aversion

**Fig. 3.**
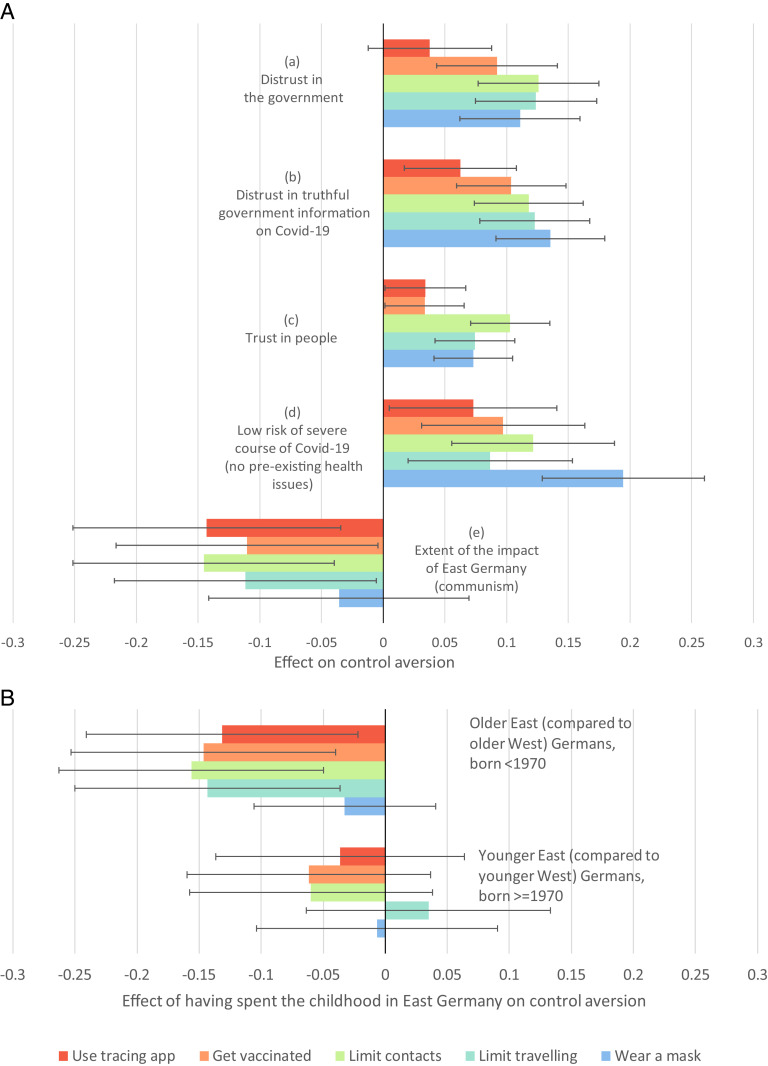
Predictors of control aversion (as measured by the difference between voluntary agreement and agreement under enforcement) for the five measures. Shown are the coefficients and 95% CI on control aversion, estimated in ordinary least squares linear regressions with standardized variables. *SI Appendix*, Tables S4 and S5 provide the full regressions. (*A*) For example, (a) shows that a SD difference in the extent to which people distrust the German government is associated with increased control aversion of somewhere between 10% and 15% of a SD for contacts, travel, and masks. (*B*) The East Germany effect reduces control aversion. The values are based on regressions identical to those of Fig. 3*A* except that the continuous variable for age is replaced by a dummy indicating born before 1970. The top group of bars shows that for all domains other than wearing a mask, older respondents from the East are about 13% to 16% of a SD less control-averse than those from the West.

What explains control aversion? The most important covariates, as shown in Fig. 3*A*, refer to (dis)trust, preexisting health issues, and experience under the coercive regime of East Germany.

The less respondents trust the German government in general (Fig. 3*A*, a), and specifically in the government’s truthful information about the coronavirus outbreak (Fig. 3*A*, b), the more control-averse they are in all five domains. Overall agreement to follow a measure (no matter if voluntary or enforced) is higher when the government is perceived as trustworthy (*SI Appendix*, Tables S6 and S7).

On the other hand, the more respondents believe that “most people” can be trusted, the more control-averse they are (Fig. 3*A*, c). This might appear contradictory at first sight. However, it is consistent with the view that trust in others reflects a belief that strong communities and social capital are sufficiently effective in ensuring compliance (14). In this framing, enforcement may be perceived as a signal of distrust by the government of its citizens, provoking a negative response (15, 18, 22). This may be one of the reasons why, when abolishing contact restrictions, the prime minister of the German state of Saxony-Anhalt stressed that “the change from enforcement to recommendation is also a sign of our trust in the population” (23).

Control aversion is stronger among healthy respondents who do not belong to the COVID-19 risk group and thus have a low risk of a severe course in case of an infection (Fig. 3*A*, d).

Finally, the more respondents were exposed to the communist regime of East Germany (as captured by the linear combination of the coefficients for having spent the childhood in a federal state belonging to East Germany and its interaction with age), the less control-averse they are (Fig. 3*A*, e). This finding is well in line with evidence from an online experiment in a very abstract setting unrelated to COVID-19 policies (17). Note that, though imprecisely estimated, this East Germany effect is substantial as it has the largest normalized effect among all variables on the tracing app and, albeit less pronouncedly, on vaccinations.

Consistent with the interpretation that the East Germany effect reflects exposure to authoritarian rule under the communist regime there, Fig. 3*B* shows that the East–West difference is qualitatively larger for older Germans who were at least 20 y old when the Berlin Wall came down (mean age today: 62.4 y) than for younger Germans who experienced less than 20 y under different regimes (mean age today: 35.3 y). Note that this age cutoff not only represents the median of the sample, but the difference between the two subsamples reflects approximately one generation. Thus, the pattern depicted in Fig. 3*B* suggests that, assuming a linear trend, the differences between East and West Germans in their responses to enforcement would take three generations to fully converge, which is in accordance with another study on the institutional effect of East Germany (24).

Older East Germans are clearly less control-averse than older West Germans in all domains except for masks. This is consistent with the effect of mere exposure (25): Those brought up in East Germany prior to 1990 were subject to ubiquitous surveillance, compulsory vaccination, and restrictions on movements (where stay-at-home orders, coming along with contact restrictions, could be interpreted as an extreme form of travel restrictions), while wearing masks is rather exotic and not part of the experience of East (and West) Germans prior to the pandemic.

This final result is remarkable as it shows that people who experienced the coercive East German regime three and more decades ago are less averse to enforced anti–COVID-19 measures today.

## What We Know about Mechanisms underlying Crowding Out

What are potential mechanisms behind crowding out in this study? People’s commitment may differ under voluntary and enforced policy implementations not only because enforcement has the power to increase cooperation but also because preferences depend on the situation, and incentives provide clues which constitute different situations (26). The fact that incentives may crowd out social preferences is an important example of such situation-dependent preferences. Three driving forces behind crowding out of social preferences have been discussed extensively in the literature, as reviewed in ref. 14.

First, implementing incentives reveals information about the policy-maker (e.g., trusting or not) and his beliefs about the target (e.g., responsible citizens or not). Eschewing enforcement in favor of voluntary anti–COVID-19 measures may signal the government’s trust in citizens’ responsible actions, while enforcement may signal the government’s belief that people cannot be trusted to be socially responsible (22), and these signals may prompt positive or negative responses to the policy.

Second, crowding out can result from moral disengagement, which occurs because incentives frame the decision problem as one in which ethical convictions are not salient. Voluntary anti–COVID-19 policies may trigger moral deliberation and convictions to be a good citizen, whereas enforcement might relieve the citizen of any need to deliberate and thus crowd out those moral convictions (27).

Third, enforcement may compromise personal autonomy (15, 18). A person’s autonomous motivation to perform an action may be undermined by inducing her to take this action as an explicit means to some extrinsic goal, such as conforming to close supervision (10, 11). This mechanism may be particularly salient because many of the anti–COVID-19 measures are far-reaching restrictions of personal freedom, privacy, and autonomy.

The autonomous motivation I address in this study is captured by internalized extrinsic motivation instead of primary intrinsic motivation (28), that is, people feel personally responsible to support a social purpose. This notion has been found to predict actual voting behavior (29) and is also plausible in the context of anti–COVID-19 policies.

## How to Limit Crowding Out of Voluntary Support for Anti–COVID-19 Policies

My results do not imply that enforcement will be ineffective; there is evidence to the contrary. According to survey vignettes conducted in Germany, the vast majority state that they would comply with a mandatory mask policy (30). My results complement these findings by showing that for other policies crowding out may be an important determinant of effectiveness that policy makers should attempt to mitigate. This may be particularly the case for policies that are intrinsically difficult to enforce.

The results in Fig. 3*A* suggest that the effectiveness of voluntary compared with enforced measures to address the COVID-19 pandemic will differ across populations. First, crowding out of intrinsic support due to enforcement may be more of a concern in liberal democracies than in populations with recent experience in coercive regimes.

Second, the more confidence people have in their government in general and in the government’s truthful information about the coronavirus outbreak the more they agree to follow both voluntary and enforced policies (*SI Appendix*, Tables S6 and S7), and the less detrimental is the effect of enforcement on voluntary support for a measure. Consistent with the literature in political science (31, 32), this suggests that enforcement might create less resistance in countries like South Korea where, according to the World Values Survey (WVS; 33), trust in government is high than in countries like Colombia where trust in government is low.

Third, policy-makers in societies where interpersonal trust and other dimensions of social capital are strong like Denmark or Norway (WVS; 33) might be particularly concerned about the adverse effects of enforcement.

Fourth, the fact that enforcement is both more accepted and also less detrimental for voluntary motivation among people with a higher risk of mortality in case of an infection suggests that mandatory measures are likely to be more effective in places like hospitals, retirement homes, or elderly communities.

## Where Enforcement May Be Limited

Some of the determinants I have just discussed are themselves subject to policy. For example, political decisions are likely to affect trust in the veracity of government’s information about the pandemic. Early attempts by governments to downplay the seriousness of the coronavirus in China (34), Brazil (35), Britain (36), and the United States (37) appear to have not only delayed the response to the pandemic, but also, using the words of CNN, “betrayed the public trust” (37). This, in turn, may cultivate citizens’ distrust in their government’s handling of the pandemic and as a result limit the efficacy of anti–COVID-19 policies when those are introduced, as my survey results suggest.

In contrast, the relative success of Germany in containing infection rates may be partly attributed to Chancellor Merkel’s scientific point of view and the public information provided by her government, notably the Robert Koch Institute’s detailed reports. The country’s leading coronavirus virologist, Christian Drosten, has been informing citizens in a regular podcast from the beginning of the pandemic in Germany, which is also likely to have increased public trust (38). My data support the potential power of such trustworthy information channels to increase compliance and reduce adverse effects of enforcement.

Is this concern with trust and control aversion misplaced? If we could be certain that enforcement would be fully effective, there might appear to be little reason to worry about crowding out. However, there are reasons to doubt both the premise and the conclusion.

First, concerning the premise, while wearing masks can be easily observed, authorities lack sufficient information to ensure citizens’ compliance for many of the measures (e.g., contact restrictions, using a contact-tracing app, social distancing, maintaining quarantine, or getting tested).

Second, the enforcement power in a liberal democracy may be limited. It would be difficult to fully enforce many of the measures without violating privacy and personal autonomy commitments, and even impossible in a country like Sweden given their constitution (39). This is perhaps a reason why, in comparison to more authoritarian regimes, democratic governments have delayed the introduction of restrictive anti–COVID-19 measures (40).

Third, the conclusion that were enforcement effective we need not worry about crowding out is not valid either. A challenge for democratic societies is the sustainability of the policies over a long period once they are effective (41). This may be impossible to achieve if enforced measures have provoked an adverse subjective response.

Beyond such limitations, enforcing behavior against people’s agreement is likely to incite discontent and aggression, which may be channeled in destructive acts such as domestic violence (42) or the riot nights in Paris, Stuttgart, Frankfurt, or Bogotá (43, 44).

## Policy Implications: Putting the Survey Results to Use

What can my data contribute to the policy discussion? The value of inferences based on a single study conducted in a single country is limited, but the urgency of the situation in many countries now experiencing a second lockdown and the intense debate about the appropriate policy response warrants at least a few speculative observations about the wisdom of enforcement for the various policy options.

Important considerations go beyond whether enforcement of a policy evokes substantial control aversion and include the level of compliance required for a policy to be successful, the share of citizens who agree to comply with a policy voluntarily, and the extent to which a policy is enforceable. Moreover, for all policies, cooperation provides positive externalities to others, while there are differences across policies and individuals in the degree to which compliance protects oneself. Below I discuss the anti–COVID-19 policies considered in this article along those lines.

In the case of wearing masks, which mainly confers significant benefits for others, a mandate is promising. Since mask compliance is easily observable and conditional cooperators (8) or conformists (45) constitute a large share of the population, compliance may not be sustainable if a substantial minority does not comply. Maintaining a compliance equilibrium requires cooperation by the large majority and thus punishment of defectors, even if their number is relatively small (45). My survey suggests that noncompliance to a voluntary mask-wearing policy would be substantial (Fig. 1). This along with the feasibility of enforcement and only moderate control aversion indicates that enforcement would be a valuable approach. Similar considerations apply to policies limiting travel, where the motivational costs of enforcement are low (Fig. 1) and enforcement can be quite effective.

The picture looks quite different for a contact-tracing app, the success of which is thought to also require compliance by a large majority (21). According to my survey results, this would be unlikely under a voluntary policy (Fig. 1). However, enforcement of a contact-tracing app would encounter significant resistance: A substantial share (40%; Fig. 2) respond negatively to enforcement, and a similar fraction shows the strongest level of opposition to enforcement (Fig. 1). Such heavy opposition evoked by a policy that is difficult to impose (e.g., people could simply leave their mobile phone at home) speaks against its enforced implementation.

With respect to vaccination, my data support voluntary regimes. According to epidemiological estimations, about two-thirds of the population need to be immune in order to reach herd immunity. This threshold may be lowered when vaccinations are not allocated randomly but primarily to those who are more likely to get infected (46). In my survey, two-thirds of Germans say they would agree to get vaccinated voluntarily (Fig. 1), whereas enforcement would evoke considerable opposition. Given my findings on trust, I suppose that citizens’ compliance will crucially depend on their trust not only in their government but especially in the particular vaccines once they are available, and therefore informing the public transparently will be essential.

While waiting for vaccines, limiting contacts is at the core of fighting the pandemic and requires extensive cooperation. As for a contact-tracing app and vaccinations, contact restrictions also evoke control aversion among more than one-third of my respondents (Fig. 2). Still, a case for some degree of enforcement can be made. Even though voluntary support is higher than support in case of enforcement, strong opposition to enforced contact restrictions is less frequent than for those other policies (Fig. 1). Voluntary cooperation of about two-thirds is unlikely to suffice, in particular as superspreading events involving younger cases have been shown to drive the pandemic to a large extent (47, 48). Therefore, some degree of enforcement may be promising to increase cooperation among healthy people who have lower incentives to avoid getting infected themselves but whose cooperation provides strong positive externalities to those at higher risk when getting infected.

Notice that the three policies for which a good case can be made in favor of enforcement—limiting contacts (especially large gatherings), masks, and travel—share a common duo of characteristics: The measures are both observable to others and require repeated cooperation. These two characteristics replicate the conditions in repeated public-goods experiments where, in the absence of punishment of free riders, cooperation unravels in successive rounds of play (6). The contact-tracing app and vaccination policies are more similar to first-round play or one-shot public-goods games for which high levels of voluntary cooperation are typically observed, with no opportunity for cooperation to decline.

Effective states govern by some combination of enforcement and voluntary compliance (49). The results of this study suggest that interventions to combat COVID-19, future pandemics, and potentially also other societal challenges such as climate change vary in the extent to which enforcement may encounter opposition that would not be provoked by appeals to voluntary participation. This may be particularly true for more invasive interventions such as contact-tracing apps and vaccinations, among people who distrust their government, and in societies with a long history of liberal democracy.

## Materials and Methods

### The Questions.

To study the possibility that enforcement may crowd out civic values it is essential not to confound social motives for adopting a measure on the one hand with obedience to the law on the other. Therefore, my questions ask about the respondent’s attitude (“agree”) toward the measure and not whether a subject would comply with a legally imposed and enforced policy. Moreover, the voluntary option in my survey has a strong normative content (“strongly recommended”). There are three reasons why the questions were formulated this way.

First, it is quite possible that people might disagree with a measure but still comply with it because they are forced to. This distinction is crucial to detect crowding out of intrinsic motivation due to enforcement in comparison with people’s agreement under voluntary policies. The answers to my questions in case of enforcement allow me to differentiate between the share of people who are comfortable (“agree”) with a measure under enforcement and are likely to comply with it and the share of citizens who disagree with it and who will therefore either not adhere to a policy even though it is mandatory, or follow it under enforcement but be left with heightened negative emotions like anger, aggression, frustration, and hostility toward their government.

Second, to investigate whether enforcement can succeed in implementing a measure (not the question I am asking), an appropriate survey question would inquire about behavior. However, a positive answer to this question is uninformative about subjects’ attitudes behind compliance. Instead, it measures the extent to which people state that they will obey the law. To understand the crowding-out phenomenon, I need to elicit people’s feelings behind their compliance behavior and not their willingness to comply with enforcement per se, which is a different research topic.

Third, the question on agreement to follow a policy in case of voluntary implementation comprises the information that the policy is strongly advised by the government. This serves to stress that even in the absence of enforcement compliance is clearly desirable. Asking the question on agreement in case of voluntary policies without stressing its normative importance would give the impression that noncompliance is equally permitted and acceptable, which is not the way in which voluntary compliance is promoted in actual policy making.

To identify the differential individual responses to enforcement and voluntary implementation, I employ a within-subjects design. Experimental findings in a setting very similar to the decision environment of my survey suggest that my results would not be substantially affected had I instead relied on a between-subjects comparison. Subjects were asked how much money they would like to transfer to another person if they are completely free in their choice and if they are forced to make a transfer. The results do not indicate any effect of confronting subjects with both possibilities or with only one alternative on subjects’ choices and the difference in choices between the two options (15).

### The Survey.

The survey questions were embedded in an ad hoc online survey on COVID-19 initiated by the Cluster of Excellence “The Politics of Inequality” at the University of Konstanz, who launched a call for survey questions. My survey questions based on my own previous work on control aversion were included in response to that call. All subjects were asked to state their agreement to follow a policy in both cases, if it remains voluntary and if it is enforced.

The sample size was predetermined by the Cluster of Excellence “The Politics of Inequality” at the University of Konstanz. Their predefined target sample size was 4,700 subjects. Participants were recruited from a commercial online access panel administered and remunerated by respondi. respondi is an online access panel where membership and participation is voluntary and follows a double opt-in registration process. Participation is incentivized with tokens which can be exchanged for goods. The online panel respondi usually does market research. Accordingly, people registered there are unlikely to have a particularly high intrinsic motivation to support science. This is important because otherwise voluntary participation in the survey might create a sample bias in favor of voluntary policies.

The survey was implemented and run by the surveyLab at the University of Konstanz from 29 April to 8 May 2020. It comprised several modules on topics related to COVID-19, with my module on agreement to follow anti–COVID-19 policies if they are voluntary or enforced being one of them. Invited participants self-selected into the online survey titled “Living in exceptional circumstances” and subjects were not aware of the specific topic of any module (including mine) when agreeing to participate.

Before and after the modules, respondents answered a series of questions on sociodemographics and other controls. Basic demographics were mandatory to answer, in particular the questions concerning the sampling criteria. All other questions remained voluntary and subjects were free to quit the survey at any time. In total, the survey contained 201 variables and median response time was 14 min.

Participants were required to be 18 y of age or older, German-speaking, and residents of Germany. The quota reflected the resident population in terms of (the marginal distributions of) age group, gender, education, and region. As a main research question of this study refers to East–West differences in reactions to enforcement and there are fewer East Germans than West Germans (12.53 million as compared to 70.64 million), double quota for East Germany were used. The mean age of the sample was 48 y (SD: 16 y) and 51% were female.

Note that all results reported in the paper and *SI Appendix* are based on unweighted observations, as the main purpose of the paper is not to make inferences about the population of Germans. The results are hardly affected and do not change qualitatively if I include sample weights computed based on the German microcensus. If at all, the unweighted results are slightly more conservative with respect to control aversion as there was oversampling for East Germans.

The following exclusion criteria were defined by the surveyLab: very high numbers of missing answers, nonsense responses to open questions, speeders, and straightlining. Exclusions were performed by the surveyLab, based on an independent standard quality check in which I was not involved in any way. Moreover, I use listwise exclusion of subjects with missing data in the variables used for the regressions. See *SI Appendix*, Table S1 for details.

This study was approved by the ethics committee of the University of Konstanz, IRB 20KN09-006. All subjects provided informed consent.

## Supplementary Material

Supplementary File

## Data Availability

The anonymized survey data and code files to replicate the results of the paper have been deposited at GESIS SowiDataNet | datorium (German Data Archive for the Social Sciences) and are available at https://doi.org/10.7802/2124 (50).

## Change History

July 7, 2021: “Article updated to correct the following sentence: “Depending on the measure, relatively few—between 12% (app) and 28% (masks)—respond positively to control, that is, their agreement is greater under compulsory instead of voluntary implementations (blue slices).” In the previous version “voluntary” and “compulsory” were inadvertently reversed in the sentence.”
