# Supplementary material for: Enforcement may crowd out voluntary support for COVID-19 policies, especially where trust in government is weak and in a liberal society
Source: Proc Natl Acad Sci U S A. 2020 Dec 21;118(1):e2016385118. doi: 10.1073/pnas.2016385118 (PMC7817206; doi:10.1073/pnas.2016385118)
Supplement: Supplementary File [file pnas.2016385118.sapp.pdf]

PNAS
www.pnas.org

Supplementary Information for

Enforcement may crowd out voluntary support for Covid19 policies, especially where trust in government is weak and in a liberal society

Katrin Schmelz

Email: katrin.schmelz@uni-konstanz.de

This PDF file includes:

Supplementary text
- Survey questions (original screenshots, in German)
- Survey questions (English translations)
- Timeline of important news on Covid-19 in Germany before and during the survey
- Validity of the survey results

Figs. S1 to S5
- Fig. S1. Timing of the survey relative to the course of the Covid-19 pandemic in German
- Fig. S2. Average agreement to follow a measure if it is voluntary or enforced.
- Fig. S3. Distributions of agreement under the voluntary vs. enforced implementation of a measure
- Fig. S4. Stability of types across the five measures
- Fig. S5. Key results of the Principal Components Analysis (PCA)

Tables S1 to S7
- Table S1. Number of participants in the survey, dropouts and exclusion criteria
- Table S2. Attempt to classify the anti-Covid-19 measures along various dimensions
- Table S3. Explanation of all independent variables used in the regressions
- Table S4. Linear regressions on control aversion for Fig. 3A
- Table S5. Linear regressions on control aversion for Fig. 3B
- Table S6. Linear regressions on voluntary agreement
- Table S7. Linear regressions on agreement under enforcement

SI References

Supplementary text

Survey questions on agreement under voluntary vs. enforced measures (original screenshots, in German)

Note: To appear natural to participants, the order of the survey questions corresponds to the sequence in which the measures they address were introduced in Germany.

Einstellungen zu verschiedenen Maßnahmen

Die Fragen auf den folgenden Seiten beziehen sich auf Maßnahmen zur Verlangsamung der Ausbreitung der Corona-Epidemie. Für jede Maßnahme bitten wir Sie anzugeben, inwieweit Sie damit einverstanden sind, wenn sie von der Regierung freiwillig oder verpflichtend eingeführt wird.

Um Ansteckungen zu verhindern, sollen die Menschen Begegnungen mit Freunden, Verwandten und Kollegen weitgehend vermeiden.

Inwieweit sind Sie einverstanden, Ihre sozialen Kontakte einzuschränken, wenn ...

	**Überhaupt nicht einverstanden 0**	**1**	**2**	**3**	**Voll und ganz einverstanden 4**
... Kontakteinschränkungen von der Regierung dringend empfohlen sind, aber freiwillig bleiben?	○	○	○	○	○
... Kontakteinschränkungen von der Regierung verpflichtend eingeführt und kontrolliert werden?	○	○	○	○	○

Weiter

Einstellungen zu verschiedenen Maßnahmen

Um Ansteckungen zu vermeiden, soll man sich möglichst zu Hause aufhalten und auch nicht verreisen.

Inwieweit sind Sie einverstanden, Ihre Reisen einzuschränken, wenn ...

	**Überhaupt nicht einverstanden 0**	**1**	**2**	**3**	**Voll und ganz einverstanden 4**
... Reisebeschränkungen von der Regierung dringend empfohlen werden, aber freiwillig bleiben?	○	○	○	○	○
... Reisebeschränkungen von der Regierung verpflichtend eingeführt und kontrolliert werden?	○	○	○	○	○

Zurück

Weiter

Einstellungen zu verschiedenen Maßnahmen

Es wird derzeit über eine App diskutiert, die auf Bewegungs- und Kontaktdaten von Mobiltelefonen zugreift, um die Benutzer anonym über eine mögliche Infektion zu informieren. Diese App ist umso nützlicher, je mehr Personen sie verwenden.

Inwieweit sind Sie einverstanden, diese App selbst zu benutzen, wenn ...

	Überhaupt nicht einverstanden 0	1	2	3	Voll und ganz einverstanden 4
... die Benutzung der App von der Regierung dringend empfohlen wird, aber freiwillig bleibt?	◯	◯	◯	◯	◯
... die Benutzung der App von der Regierung verpflichtend eingeführt und kontrolliert wird?	◯	◯	◯	◯	◯

Zurück Weiter

Einstellungen zu verschiedenen Maßnahmen

Wenn es einen zugelassenen Impfstoff gegen das Coronavirus gibt:

Inwieweit sind Sie einverstanden, sich selbst impfen zu lassen, wenn ...

	Überhaupt nicht einverstanden 0	1	2	3	Voll und ganz einverstanden 4
... die Impfung von der Regierung dringend empfohlen wird, aber freiwillig bleibt?	◯	◯	◯	◯	◯
... die Impfung von der Regierung verpflichtend eingeführt und kontrolliert wird?	◯	◯	◯	◯	◯

Zurück Weiter

Einstellungen zu verschiedenen Maßnahmen

Es wurde lange über das Tragen von Masken in der Öffentlichkeit diskutiert, wo der Mindestabstand von 1,5m nicht eingehalten werden kann.

Inwieweit sind Sie einverstanden, selbst eine Maske zu tragen, wenn ...

	Überhaupt nicht einverstanden 0	1	2	3	Voll und ganz einverstanden 4
... das Tragen von Masken von der Regierung dringend empfohlen wird, aber freiwillig bleibt?	◯	◯	◯	◯	◯
... das Tragen von Masken von der Regierung verpflichtend eingeführt und kontrolliert wird?	◯	◯	◯	◯	◯

Zurück Weiter

Survey questions on agreement under voluntary vs. enforced measures (English translations)

Attitudes to different measures

The questions on the following pages refer to measures to slow down the spread of the corona epidemic. For each measure, we ask you to indicate to what extent you agree with it if it is introduced by the government voluntarily or compulsorily.

[Limit contacts]

To prevent infection, people should avoid meeting friends, relatives and colleagues as far as possible.
<u>To what extent do you agree to limit your social contacts if ...</u>
… contact restrictions are strongly recommended by the government, but remain voluntary?
… contact restrictions are made mandatory and checked by the government?
(Answers to all questions are provided on a 5 point Likert scale, ranging from 0 “not agree at all” to 4 “fully agree”.)

[Limit travelling]

In order to avoid infection, one should preferably stay at home and also not travel.
<u>To what extent do you agree to limit your travelling if ...</u>
... travel restrictions are strongly recommended by the government but remain voluntary?
... travel restrictions are imposed and checked by the government?

[Wear a mask]

There has been a long discussion about wearing masks in public where the minimum distance of 1.5m cannot be maintained.
<u>To what extent do you agree to wear a mask yourself if ...</u>
… wearing masks is strongly recommended by the government but remains voluntary?
… wearing masks is made mandatory and checked by the government?

[Use tracing app]

We are currently discussing an app that accesses the movement and contact data of mobile phones to inform users anonymously about a possible infection. This app is more useful the more people use it.
<u>To what extent do you agree to use this app yourself if ...</u>
… using the app is strongly recommended by the government but remains voluntary?
… using the app is made mandatory and checked by the government?

[Get vaccinated]

If there is an approved vaccine against the coronavirus:
<u>To what extent do you agree to be inoculated yourself if ...</u>
… vaccination is strongly recommended by the government but remains voluntary?
… vaccination is made mandatory and checked by the government?

Timeline of important news on Covid-19 in Germany before and during the survey

January 2020

27 January. First German infected with Covid-19.

February 2020

24 February. Covid-19 has arrived in Europe as an epidemic - serious situation in Italy.

26 February. First episode of the podcast "The coronavirus update" with the Head of Virology at the Berlin Charité and developer of a widely used PCR test, Christian Drosten. He informs the German population about scientific insights on the coronavirus and discusses the potential benefit of anti-Covid-19 measures. It is broadcasted over several months, several times per week, and will be followed by millions of Germans.

March 2020

2 March.
- Recommendations: sneeze into the arm bends, wash your hands regularly, avoid handshakes.
- Export of medical protective equipment (breathing masks, gloves, protective suits, etc.) abroad is prohibited.

10 March. Recommendation: cancellation of all major events with more than 1,000 participants.

16 March. Press conference by Chancellor Merkel on anti-Covid-19 measures of the Federal Government, announcing additional measures to reduce social contacts, e.g.:
- Schools and day-care centers are already closed in most of the federal states, others will follow.
- Numerous shops are to close – except for supermarkets, pharmacies, drugstores, petrol stations and hairdressers.
- Restaurants may only open between 6am and 6pm.
- Places of worship, playgrounds, sports facilities, bars, clubs, theatres, cinemas, concert halls and museums will be completely closed.
- Restrictions on travel, borders are closed.

18 March.
- Historical TV address of Chancellor Merkel. In an urgent appeal, she calls on the population to act in solidarity and responsibility. "Social contacts must be minimized." "This is serious. Take it seriously, too."
- EU imposes entry ban.
- Robert-Koch-Institute (RKI): coronavirus vaccine realistic in spring 2021.

20 March. Bavaria imposes state-wide curfew.

22 March.
- Federal and state governments agree on strict restrictions on exit and contact. Citizens may only be in public areas with a maximum of one person who does not live in the same household and must keep at least 1.5m distance from others.
- Restaurants and pubs may only offer take-away food. Hairdressers must close.

27 March. Discussion about pros and cons of wearing face masks.

28 March. Infection Protection Act comes into force (i.e., the government is entitled to restrict fundamental rights).

31 March. The city of Jena introduces mandatory mask-wearing in public areas. Other cities are more skeptical.

April 2020

1 April.

- The nationwide contact restrictions are extended until 19 April. People should generally refrain from private travel and visits - including those by relatives.
- 130 scientists and entrepreneurs present a concept for a corona-app “PEPP-PT”. With this concept, countries should be able to build data protection-friendly apps across Europe that work across borders.

3 April.

- A total of 37 million protective masks have arrived in Germany.
- Drosten reports about a new Science publication on the benefits of corona-apps *(12)* in his podcast.

6 April. Corona-app ready for use in the next days or weeks. Such a tracing app is part of the exit strategy of the federal government in order to gradually loosen the contact bans and other restrictions.

15 April.

- The severe restrictions on contact will be extended until 3 May.
- Stepwise reopening of schools on May 4.
- Shops with a sales area of up to $800m^2$ will be allowed to reopen from 20 April, subject to conditions relating to hygiene, access control and queuing.
- Restaurants, bars and pubs are to remain closed as before.
- Major events will also remain prohibited until at least 31 August.

16 April. Government recommends to wear community masks when shopping and in public transport.

17 April. Germany survived the first wave of Covid-19 well, gradually returning to normality.

20 April.

- First cautious relaxations of the anti-Covid-19 measures come into force. Many federal states again allow shopping in stores up to $800m^2$.
- Open letter of 300 researchers criticizing the centralized approach of the corona-app “PEPP-PT” because of data protection issues.

21 April. The worldwide travel warning of the German government still applies, at least until mid-June.

22 April. Paul-Ehrlich-Institute has approved the first clinical trial of a corona vaccine to be tested in Germany.

23 April. Debate about how useful masks are.

26 April.

- Minister of Health Spahn pleads for corona app, which should be installed on as many mobile phones as possible. It should help to track when infected people meet others and warn these contact persons.
- The German government has made an important decision on the direction of its planned corona warning app. It is moving towards decentralized storage of user data. According to experts, this solution is better for data protection than a centralized data reconciliation. It also clears the way for linking the apps with the smartphone systems of Apple and Google. This should make the apps more efficient and secure.

27 April. Wearing face masks is mandatory in all federal states for shopping and public transport.

SURVEY STARTS ON 29 APRIL

29 April.

- The German government has extended the worldwide travel warning for tourists until at least 14 June.
- Interior Minister Seehofer wants to extend the controls at German borders until 15 May.
- First test subjects of German vaccination study have been injected.

30 April. Chancellor Merkel is consulting with the heads of the federal states on how to proceed in the Covid-19 crisis:

- Contact restrictions remain in force for the time being. Citizens are to keep a minimum distance of 1.5 meters in public and only stay there alone, with another person not living in the household or with members of their own household.
- Playgrounds are to be permitted again under certain conditions. The final decision on whether to open them would be made by the federal states.
- Museums, zoos, exhibitions and memorials should also be allowed to open again.
- Community worship services should be allowed again with rules on distance and hygiene.
- Schools and daycare centers: no changes, federal and state governments want to discuss this in more detail on 6 May.
- Restaurants, hotels and cafés will remain closed.
- No changes for the time being with respect to store openings.

May 2020

2 May.

- Some federal states relax some measures, contrary to the federal and state agreements from 30 April.
- Demonstrations against the corona restrictions.

4. May. Hairdressers reopen.

5. May. RKI head Lothar Wieler pleads for different relaxations of the corona measures in Germany depending on the location. The decisive factor should be the occurrence of infections.

6. May. The federal and state governments have agreed on further relaxations, though contact restrictions remain largely in place:

- Families from two different households can meet.
- Schools: gradual reopening.
- Daycare: remain closed.
- Hotels and restaurants: gradual reopening, subject to hygiene and distancing conditions.
- Shops: all shops can open but have to meet hygiene and distancing requirements. Wearing masks is mandatory.
- Corona warning app: double voluntariness is emphasized concerning its use and data transfer. A concrete date for the introduction of the app is still not mentioned.
- Districts with more than 50 infections per 100,000 inhabitants within the last 7 days will have to return to stricter restrictions immediately.

7. May. Protests among politicians against the extension of border controls by Federal Interior Minister Seehofer.

8. May.

- Three districts already break the negotiated upper limit for new infections.
- Growing resistance against the corona measures. Critics say that the restrictions of basic rights are too severe. Many even fear a compulsory vaccination - for a corona vaccine that does not yet exist.

SURVEY ENDS ON 8 MAY

Validity of the survey results

The fact that my survey responses map the actual take-up of the app in Germany suggests substantial validity. There is further evidence in support of the validity of the survey findings. In the following, I first report on neuropsychological evidence showing that control aversion is a stable psychological phenomenon. Then I show that important determinants of control aversion identified in this survey are consistent with other empirical studies.

Neuropsychological evidence has shown that the heterogeneity in control aversion across people is reflected in systematic differences of the stable functional brain organization. A neural trait underlying control-averse behavior has been identified, i.e., a task-independent neural measurement that is stable across time, similar to a neural fingerprint (1). My discussions on types in the article and the principal component analyses suggesting fairly correlated responses to enforcement across policy interventions (see figures S4 and S5 for details) are in line with this observation.

Important determinants of control aversion identified in this survey have also been found in an incentivized online experiment where control aversion was studied in a very abstract principal-agent setting (2). First, the survey finding that those raised in East Germany under communist rule are less averse to enforcement regarding anti-covid-19 policies than West Germans of the same generation is well in line with behavior in this online experiment. That experiment also finds convergence of East-West differences in younger generations.

Second, the survey finding on the belief that most people can be trusted increasing control aversion has also been observed in the online experiment just mentioned, which shows that the more people believe in trustworthiness of strangers, the more control averse they behave.

Moreover, the finding that distrust in government is associated with control aversion is consistent with the idea that a negative view of the purposes of an intervention contributes to a control averse reaction (3). In an incentivized laboratory experiment, control averse behavior is less among those who say that they understand the principal's action (4). Negative responses to enforcement may not be stimulated by control *per se* but instead arise from a lack of understanding of its beneficial purpose (5). Consistent with this view, negative feelings about the principal (here, the government) implementing control may nurture control aversion, possibly explaining my survey result that confidence in the government and its truthful information about the pandemic decreases reported control aversion.

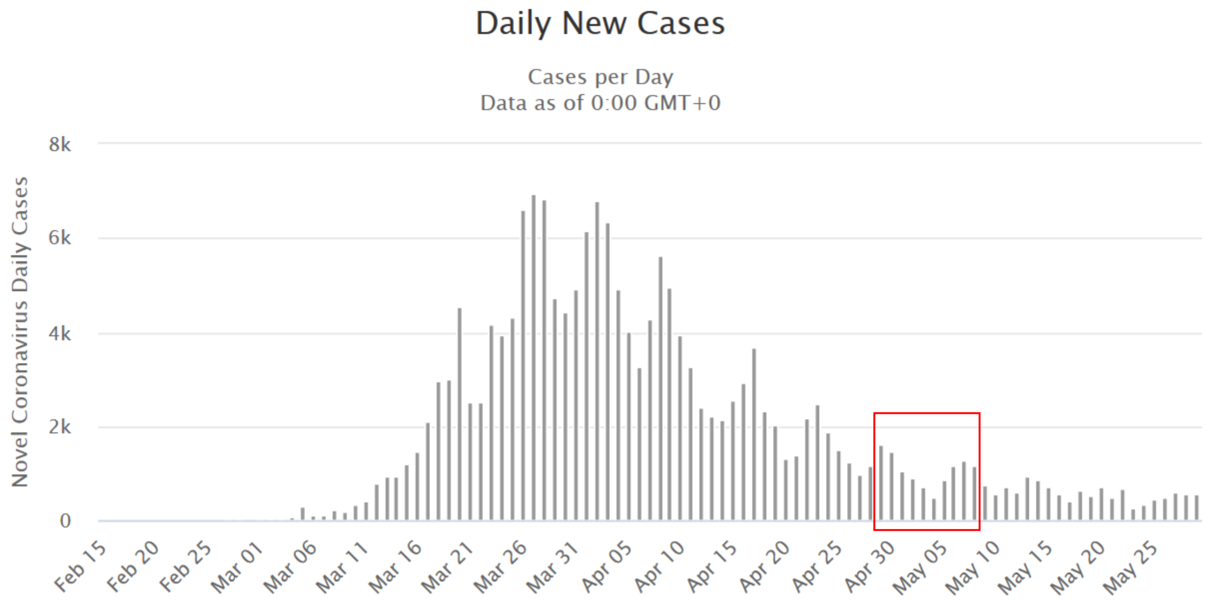

Fig. S1. Timing of the survey relative to the course of the Covid-19 pandemic in Germany. The red frame indicates the time window when the survey was conducted. The chart is taken from worldometers.org (6).

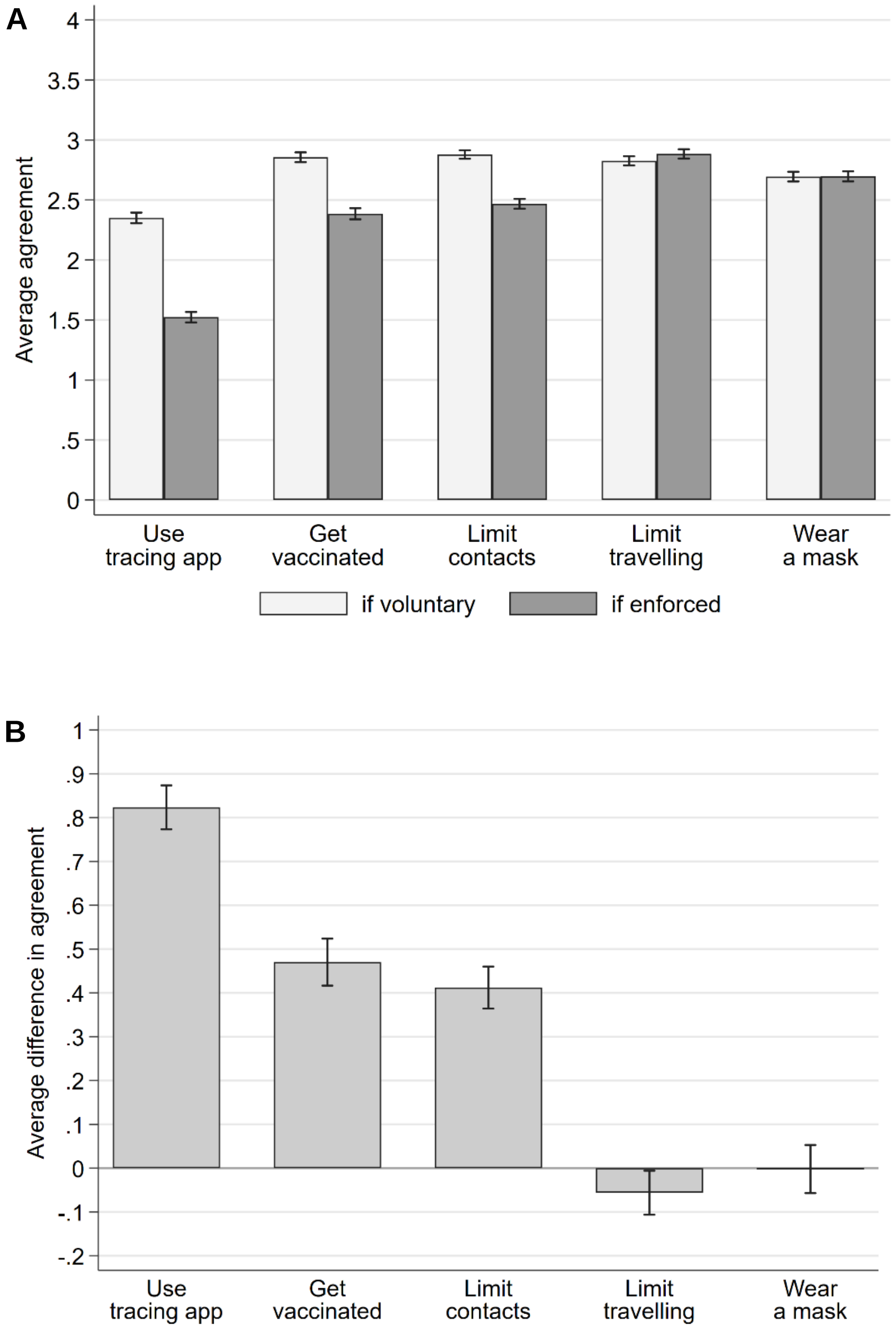

Fig. S2. (A) Average agreement to follow a measure if it is voluntary or enforced. The sample sizes correspond to Fig. 1. **(B)** Average difference in agreement in case of voluntary versus enforced implementation of a policy (in Likert scale units). The sample sizes correspond to Fig. 2. In both charts, error bars represent 95% CI.

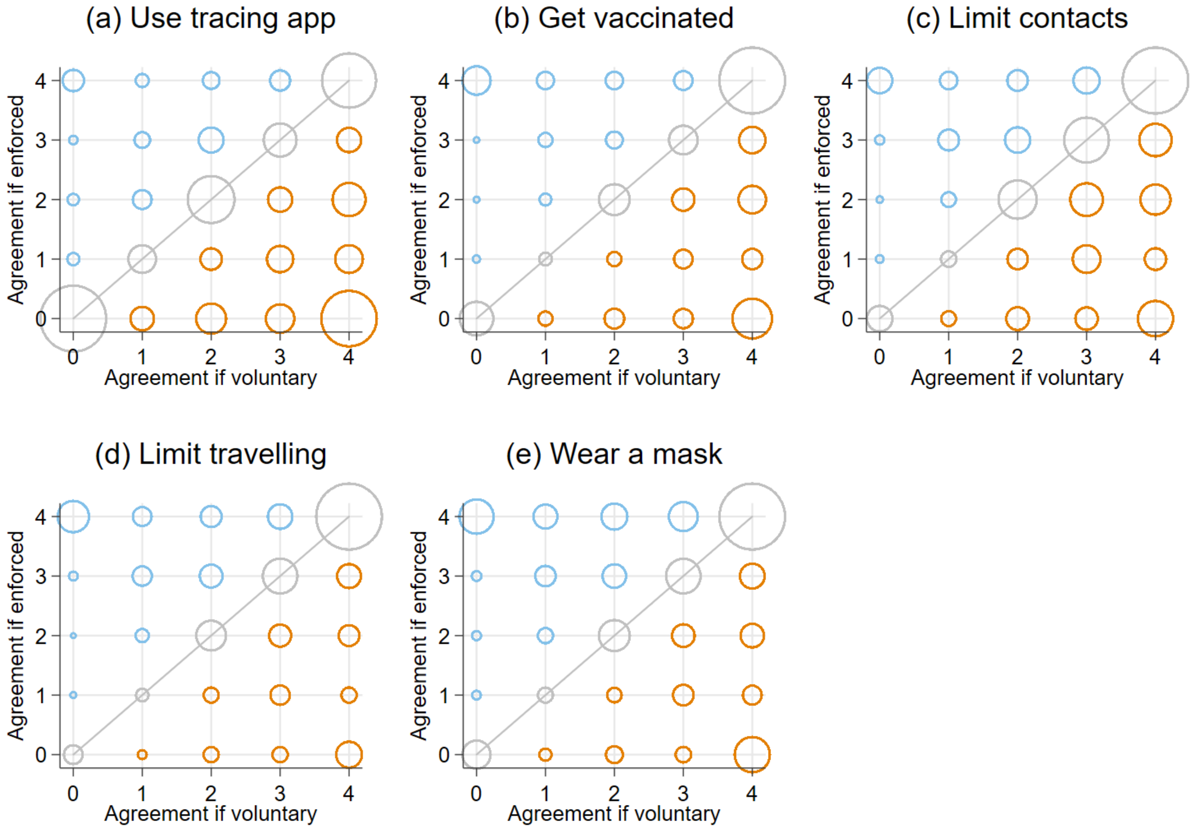

Fig. S3. Distributions of agreement under the voluntary vs. enforced implementation of a measure. The sample sizes are shown in Fig. 2. The size of a bubble increases with the frequency of respondents. Bubbles on the grey line represent neutral responses to enforcement, i.e., an individual's voluntary agreement equals his agreement if enforced. Blue bubbles above the diagonal represent individuals who increase their agreement under enforcement. Orange bubbles below the diagonal refer to control averse individuals whose voluntary agreement is higher than their agreement if controlled.
Note: Anti-Covid-19 measures are largely endorsed in the German population as a substantial share of respondents state highest agreement under both voluntary and mandatory implementations. Control aversion (orange bubbles) is ubiquitous and always at least as frequent as positive responses to enforcement. Participants who increase their agreement to a measure under enforcement (blue bubbles) appear most frequently in the mask domain (e). The grey bubbles on the diagonal account for the roughly 50% of the population who is neutral with respect to the implementation scheme. Overall agreement is lowest for the contact tracing app (a). Still, control aversion is most severe in this domain.

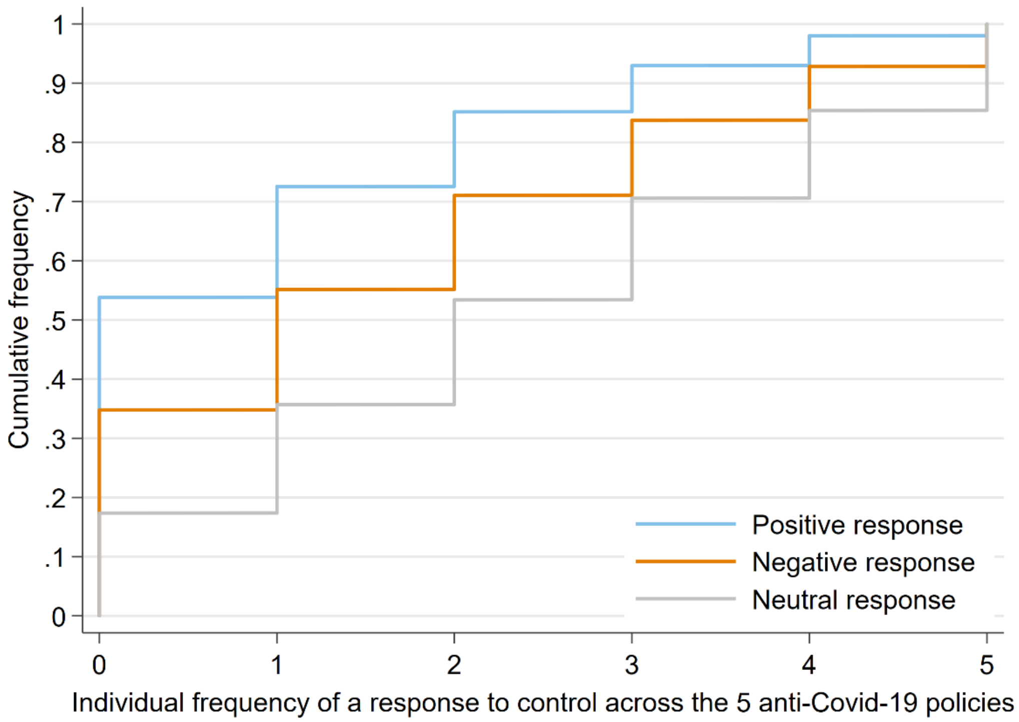

Fig. S4. Frequency of responses to enforcement across the five anti-Covid-19 policies (app, vaccination, limiting contacts, limiting travels, wearing masks). For each survey respondent (n = 4,796), I count the number of policies for which her/his response to control is negative (agreement if enforced < agreement if voluntary), neutral (agreement if enforced = agreement if voluntary), and positive.

Note: Control aversion, i.e., negative responses to control (agreement if enforced < agreement if voluntary, orange line), can be associated with a relatively persistent type. 65% are control averse with respect to at least one policy, 29% are control averse on the majority (i.e., at least three) of the policies, and 7% show control averse responses for all five measures.

Positive responses to control (agreement if enforced > agreement if voluntary, blue line) are rare. Only 46% respond positively to control at least once, 15% do so for the majority of measures, and 2% always respond positively to enforcement.

The type of control neutral respondents (agreement if enforced = agreement if voluntary, grey line) is most common and fairly robust. 83% show a neutral response at least once, 47% do so for the majority of measures, and 15% always respond neutrally to enforcement.

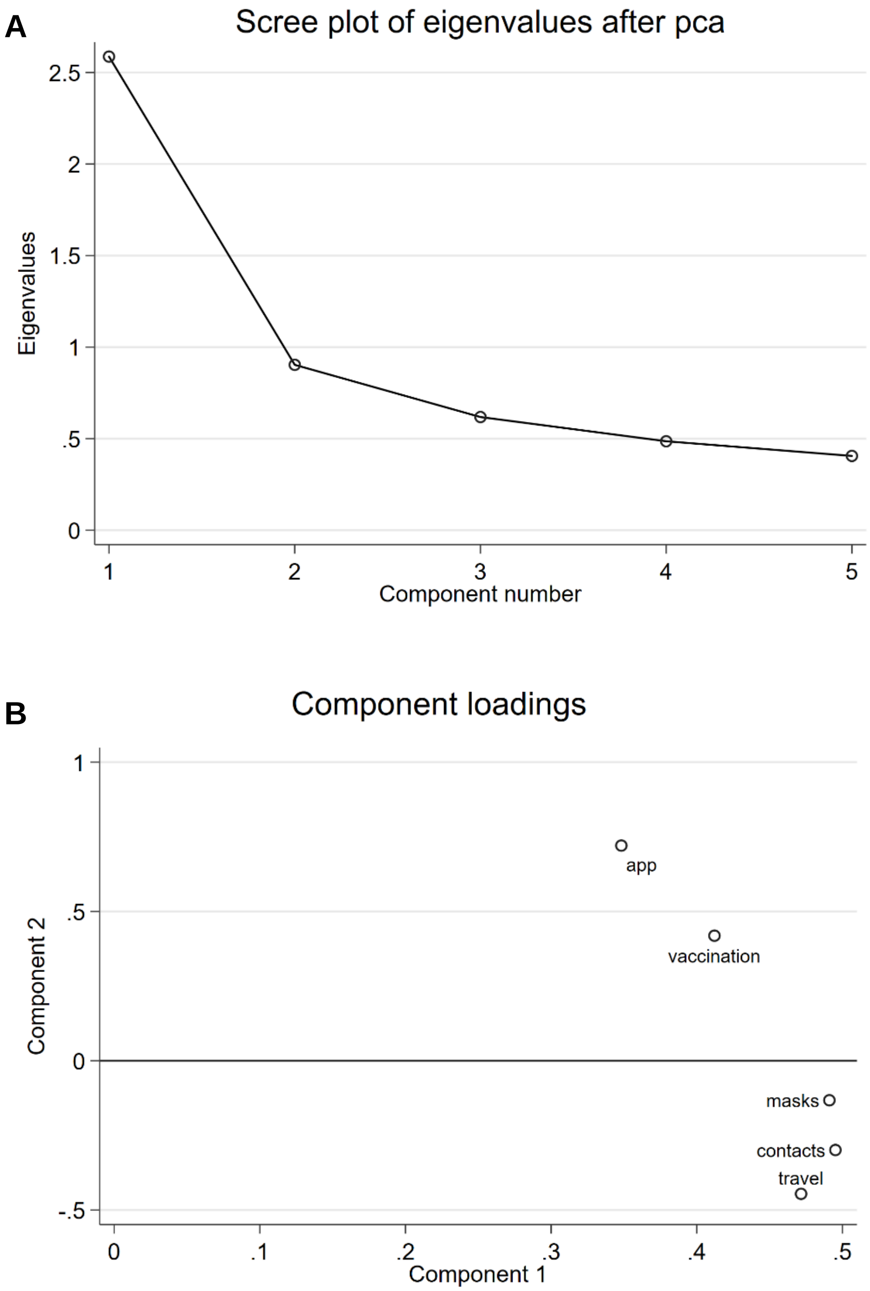

Fig. S5. Key results of the Principal Components Analysis (PCA). Variables are the differences between voluntary agreement and agreement under enforcement across the anti-Covid-19 measures (n = 4,722). **(A)** The scree plot shows the eigenvalues of all components. **(B)** The loading plot shows the loadings of the five measures on the first two components.

Note: According to **(A)**, only the first two components are important enough to consider as the curve flattens after the second component and the eigenvalues of the remaining components are far below 1.

(B) visualizes that all policies have large and similar positive loadings on the first component. Contact tracing app and vaccination have large positive loadings on the second component, while the other three domains load negatively on component 2.

Table S1. Number of participants in the survey, dropouts and exclusion criteria. Exclusions according to the five criteria in the upper part of the table were performed by the surveyLab, based on an independent quality check.

	**Number of participants**
Survey started	6,340
Dropout at the very beginning of the survey (compulsory socio-demographic information incomplete or dropout after the first module with [almost] no valid answers; my module was placed second)	1,032
Very high number of missing answers	241
Nonsense responses to open questions	36
Speeders who completed the survey in less than 6min (median 14min, reasonable answers impossible below 6min)	144
Straightlining (same responses across various question blocks)	88
**Basis for the article (Figs. 1-2 and PCA)**	**4,799**
Childhood neither in East nor in West Germany (or missing value)	105
Missing values for other variables used in the regressions on control aversion	421 (contact) to 436 (mask)
**Used in the regressions (Fig. 3)**	**4,258 (mask) to 4,273 (contact)**

Table S2. Attempt to classify the anti-Covid-19 measures along various dimensions.

	**Anti-Covid-19 measure**				
**Dimension**	Use tracing app	Get vaccinated	Limit contacts	Limit travelling	Wear a mask
Privacy intrusion	✓	✓	✕	✕	✕
Uncertainty about personal negative consequences	✓	✓	✕	✕	✕
Visible (social control)	✕	✕	✓	✓	✓
Requires repeated cooperation	✕	✕	✓	✓	✓
Concrete (vs. abstract)<br>(Construal Level Theory (7))	✕	✕	✓	✓	✓
Implemented at the time of the survey<br>(Description Experience Gap (8))	✕	✕	✓	✓	✓
Fully reversible (no long-term effects once the measure is abolished)	✕	✕	✓	✓	✓
Fully enforceable	✕	✕	✕	✓	✓
Not doing sth. we are used to do	✕	✕	✓	✓	✕
Doing sth. we are not used to do	✓	✓	✕	✕	✓

Note: These dimensions and classifications are of speculative nature, originating from my own reasoning and the reasoning of my colleagues who have kindly commented on the paper. For some of the domains, the classification provided is debatable and there might be heterogeneity with respect to their perception.

Table S3. Explanation of all independent variables used in the regressions. Q1 refers to the first quartile, Q2 refers to the second quartile (median), and Q3 refers to the third quartile. Variables referred to in Fig. 3 of the article are highlighted in dark blue.

Variable	Description and remarks	Survey question	Distribution
East_childhood	Dummy variable taking the value one if a person spent her or his childhood in a federal state of East Germany. It takes the value zero for West Germany and is missing otherwise.	In which federal state did you spend most of your childhood?	29% East, 69% West, 2% missing (n=105).
Age	In years at the time of the survey, computed from year of birth.		Mean=48.43 years, SD=15.95, Q1=35, Q2=50, Q3=61.
*East_childhood + East_childhood*Age*	Captures "the extent of the impact of East Germany (communism)". Results from table S4 are shown in Fig. 3A (e).		
Born<1970	Dummy variable taking the value one for people born before 1970 and zero for people born in 1970 or later.		49% born before 1970, 51% born in/after 1970.
*East_childhood + East_childhood*Born<1970*	Refers to older East (compared to older West) Germans who were born before 1970. Results from table S5 are shown in Fig. 3B.		
Trust_gov	Inverse of the results from table S4 are shown in Fig. 3A (a).	Here you can now see a number of public bodies and institutions. How much confidence do you generally have in them? Federal government Experts from science Media (7-point Likert scale ranging from "no confidence at all" to "a great deal of confidence") (9)	Mean=4.16, SD=1.88, Q1=3, Q2=5, Q3=6, missing: n=21.
Trust_science			Mean=4.69, SD=1.71, Q1=4, Q2=5, Q3=6, missing: n=12.
Trust_media			Mean=3.36, SD=1.65, Q1=2, Q2=3, Q3=5, missing: n=12.
Cov19_truth_gov	Treated as a continuous measure by assigning values from 1 to 5 to the response categories. Inverse of the results from table S4 are shown in Fig. 3A (b).	How factually truthful do you think your country's government has been about the coronavirus outbreak? (very untruthful – somewhat untruthful - neither nor - somewhat truthful - very truthful) (10)	Mean=3.27, SD=1.16, Q1=2, Q2=4, Q3=4, missing: n=14.

Trust_people	Inverse of the results from table S4 are shown in Fig. 3A (c).	Generally speaking, would you say that most people can be trusted or that you can't be too careful in dealing with people? (11-point Likert scale ranging from 0 "can't be too careful" to 10 "most people can be trusted") (9)	Mean=4.53, SD=2.77, Q1=2, Q2=5, Q3=7, missing: n=40.
Cov19_risk_group	Dummy indicating whether a person would be at high risk when infected with Covid-19.<br>Inverse of the results from table S4 are shown in Fig. 3A (d).	Do you belong to the Covid-19 risk group due to pre-existing health issues? (yes/no)	62% risk group, 37% no risk group, 1% missing (n=65).
Cov19_totalN	Total (cumulative) number of Covid-19 cases registered in the federal state of the participant's current residence at the day prior to survey participation, as published by the RKI (Robert-Koch-Institute, German government's central scientific institution responsible for diseases, (11)). This is the measure which has been most prominently communicated to the public.		Mean=18,216, SD=15,645, Q1=4,604, Q2=10,067, Q3=33,216.
Cov19_decline	Decline of daily new Covid-19 cases over the month before the survey in the federal state of the participant's current residence.<br>It is captured by the difference of daily infections, relying on 5-days-averages, between 1 April ±2 days (around the peak of the first wave in Germany) and 1 May ±2 days (around the beginning of the survey, see fig. S1). The data are taken from the RKI (11).		Mean= 479, SD=445, Q1=103, Q2=255, Q3=792.
Date_time	Timestamp of survey participation, in seconds.		To give an impression of the distribution, I reduce the information in this variable to the 9 days of the survey. Mean=4.74, SD=2.07, Q1=3, Q2=5, Q3=6.
Female	Dummy variable which takes the value one for female and zero for male.		51% female, 49% male.
Education_middle	Dummy for intermediate levels of schooling.	What is your highest school leaving certificate? (subjects choose from a list of options)	31% low, 33% middle, and 36% high education.
Education_high	Dummy for high levels of schooling.		

Household_income	Continuous measure of household income (in euros).	What was the total net income of the household you live in per month [before the crisis]? (Wage, salary, pension, child allowance or other income after deduction of taxes and social security. If you do not know the exact income, please estimate the monthly amount.)	Mean= 2866 euros, SD=5974, Q1=1400, Q2=2300, Q3=3500, missing: n=281.
Political_right	Continuous measure of a person' political orientation from left to right.	In politics one sometimes speaks of "left" and "right". Where on this scale would you rate yourself if 0 represents left and 10 represents right? Which number fits your position best? (11-point Likert scale)	Mean=5.64, SD=3.01, Q1=4, Q2=5, Q3=7, missing: n=18.
Voter	Dummy indicating some kind of civicness (12). It takes the value one for those who stated to have voted in the last election and zero otherwise.	Did you vote for the last federal election in 2017? (Yes / no / I don't know)	82% voted, 17% did not vote/did not know, 1% missing (n=37).
Single_household	Dummy which indicates whether the person lives alone. It takes the value zero if more than one person live in the respondent's household.	How many people currently live in your household, including yourself?	28% single households, n=0 missing (answer was mandatory).
Urban	Though this is an ordinal measure, for simplicity, I treat it as a continuous variable (with 1 being "a village or rural area" and 4 being "a big city"). The regression results are virtually unaffected if I use a dummy for each category instead.	What best describes the area in which you live? Is that...<br>(A big city;<br>A suburb or outskirts of a large city;<br>A town or small town;<br>A village or rural area)	30% big city, 13% suburb, 34% town, 23% rural, 0.4% missing (n=18).
Religious	Continuous measure	One can also be religious without belonging to a religious community. Please imagine a scale from 0 to 10. 0 means that you are "not religious at all", 10 means that you are "very religious". Where would you place yourself?<br>(11-point Likert scale)	Mean=3.15, SD=3.21, Q1=0, Q2=2, Q3=6, missing: n=41.

Table S4. Linear OLS regressions on control aversion for Fig. 3A, with the continuous variable *Age* and the interaction term *East_childhood*Age*. All dependent and independent variables are standardized (except for dummies). See table S3 for explanations of the independent variables. Coefficients of variables (or their linear combinations) shown in Fig. 3A are highlighted in dark blue.

	**Dependent variable: control aversion (agreement if voluntary – agreement if enforced)**				
	Use tracing app	Get vaccinated	Limit contacts	Limit travelling	Wear a mask
East_childhood	-0.0811**	-0.0975**	-0.105***	-0.0507	-0.0222
	(0.0411)	(0.0402)	(0.0401)	(0.0403)	(0.0399)
Age	0.00761	-0.0187	0.0262	0.0543**	0.0444**
	(0.0224)	(0.0219)	(0.0218)	(0.0220)	(0.0217)
*East_childhood*Age*	-0.0620*	-0.0128	-0.0401	-0.0610*	-0.0137
	(0.0353)	(0.0345)	(0.0345)	(0.0346)	(0.0343)
Trust_gov	-0.0378	-0.0922***	-0.126***	-0.124***	-0.111***
	(0.0256)	(0.0250)	(0.0249)	(0.0251)	(0.0248)
Trust_science	0.0287	-0.0564***	0.00515	-0.0193	-0.0391*
	(0.0214)	(0.0210)	(0.0209)	(0.0211)	(0.0208)
Trust_media	-0.0466**	-0.0431**	-0.0500**	-0.0244	-0.0344*
	(0.0205)	(0.0200)	(0.0200)	(0.0201)	(0.0199)
Cov19_truth_gov	-0.0625***	-0.104***	-0.118***	-0.123***	-0.135***
	(0.0232)	(0.0227)	(0.0226)	(0.0227)	(0.0225)
Trust_people	0.0341**	0.0336**	0.103***	0.0742***	0.0730***
	(0.0167)	(0.0163)	(0.0163)	(0.0164)	(0.0162)
Cov19_risk_group	-0.0728**	-0.0971***	-0.121***	-0.0866**	-0.194***
	(0.0346)	(0.0337)	(0.0336)	(0.0339)	(0.0335)
Cov19_totalN	0.107	0.0277	-0.0947	-0.0839	0.0102
	(0.0934)	(0.0913)	(0.0910)	(0.0917)	(0.0904)
Cov19_decline	-0.156*	-0.0590	0.0340	0.0446	-0.0701
	(0.0927)	(0.0907)	(0.0904)	(0.0910)	(0.0898)
Date_time	0.00535	0.0411***	0.0420***	0.0386**	0.00577
	(0.0160)	(0.0156)	(0.0156)	(0.0157)	(0.0155)
Female	-0.0899***	0.0458	-0.0185	0.0337	-0.0886***
	(0.0315)	(0.0308)	(0.0307)	(0.0309)	(0.0306)
Education_middle	-0.0330	-0.00135	-0.0434	0.0525	0.00298
	(0.0422)	(0.0412)	(0.0412)	(0.0414)	(0.0409)
Education_high	-0.114**	0.0391	-0.0473	0.128***	0.0212
	(0.0451)	(0.0441)	(0.0440)	(0.0443)	(0.0437)
Household_income	-0.0256*	-0.00989	-0.0136	-0.00316	-0.0195
	(0.0151)	(0.0147)	(0.0147)	(0.0148)	(0.0146)
Political_right	-0.0170	-0.000503	-0.0122	-0.0231	-0.00885
	(0.0163)	(0.0159)	(0.0159)	(0.0160)	(0.0158)
Voter	0.0472	0.0451	0.0478	-0.0120	-0.00930
	(0.0437)	(0.0428)	(0.0427)	(0.0430)	(0.0424)
Single_household	0.0924***	0.0666**	0.0774**	0.0427	0.0467
	(0.0346)	(0.0338)	(0.0337)	(0.0339)	(0.0335)
Urban	0.0126	0.00911	0.00695	0.00546	0.0252
	(0.0159)	(0.0155)	(0.0155)	(0.0156)	(0.0154)
Religious	-0.0459***	-0.00360	-0.00852	-0.0132	-0.0194
	(0.0163)	(0.0159)	(0.0159)	(0.0160)	(0.0158)
Constant	0.0814	-0.0228	0.0632	-0.0283	0.120**
	(0.0520)	(0.0509)	(0.0507)	(0.0510)	(0.0504)
Observations	4,260	4,265	4,273	4,270	4,258
R-squared	0.025	0.067	0.068	0.064	0.085

(Standard errors in parentheses) *** p<0.01, ** p<0.05, * p<0.1

Note: The linear combination "*East_childhood* + *East_childhood * Age*" refers to "the extent of the impact of East Germany" in Fig. 3A (e).

Other predictors in addition to those discussed in the paper perform as follows: (i) control aversion largely increases over the 10 days of the survey, which was at a time when measures were relaxed; (ii) females are less control averse than males concerning apps and masks; (iii) high (compared to low) education predicts less control aversion concerning apps and more control aversion with respect to limitations on travel; (iv) trust in science reduces control aversion concerning vaccination; (v) trust in the media reduces control aversion; (vi) people living alone are more control averse concerning the app, vaccination, and contact limitations; (vii) more religious people are somewhat less control averse, particularly concerning the use of the app; and (ix) none of the variables on the numbers of registered Covid-19 infections or their decline, political orientation, household income, living in a rural or urban area, or having voted in the previous national election (as a proxy for civicness) has a meaningful impact on control aversion.

Table S5. Linear OLS regressions on control aversion for Fig. 3B, with the dummy variable *Born<1970* and the interaction term *East_childhood*Born<1970*. All dependent and independent variables are standardized (except for dummies). See table S3 for explanations of the independent variables. Coefficients of variables (or their linear combinations) shown in Fig. 3B are highlighted in dark blue.

	**Dependent variable: control aversion (agreement if voluntary – agreement if enforced)**				
	Use tracing app	Get vaccinated	Limit contacts	Limit travelling	Wear a mask
East_childhood	-0.0363	-0.0615	-0.0599	0.0348	-0.00675
	(0.0512)	(0.0500)	(0.0499)	(0.0502)	(0.0496)
Born<1970	0.00840	0.0244	0.0846**	0.125***	0.0805*
	(0.0427)	(0.0417)	(0.0416)	(0.0419)	(0.0414)
*East_childhood*Born<1970*	-0.0952	-0.0851	-0.0965	-0.178***	-0.0258
	(0.0683)	(0.0668)	(0.0666)	(0.0670)	(0.0662)
Trust_gov	-0.0379	-0.0925***	-0.126***	-0.123***	-0.110***
	(0.0256)	(0.0250)	(0.0249)	(0.0251)	(0.0248)
Trust_science	0.0289	-0.0551***	0.00449	-0.0203	-0.0409**
	(0.0214)	(0.0210)	(0.0209)	(0.0210)	(0.0208)
Trust_media	-0.0461**	-0.0447**	-0.0498**	-0.0236	-0.0332*
	(0.0205)	(0.0200)	(0.0199)	(0.0201)	(0.0198)
Cov19_truth_gov	-0.0630***	-0.104***	-0.118***	-0.123***	-0.135***
	(0.0232)	(0.0227)	(0.0226)	(0.0227)	(0.0225)
Trust_people	0.0338**	0.0328**	0.102***	0.0738***	0.0732***
	(0.0167)	(0.0163)	(0.0163)	(0.0164)	(0.0162)
Cov19_risk_group	-0.0740**	-0.109***	-0.128***	-0.0857**	-0.192***
	(0.0342)	(0.0334)	(0.0333)	(0.0336)	(0.0332)
Cov19_totalN	0.107	0.0246	-0.0949	-0.0834	0.0125
	(0.0934)	(0.0913)	(0.0910)	(0.0916)	(0.0904)
Cov19_decline	-0.157*	-0.0537	0.0353	0.0440	-0.0727
	(0.0927)	(0.0907)	(0.0903)	(0.0910)	(0.0898)
Date_time	0.00582	0.0452***	0.0434***	0.0373**	0.00372
	(0.0158)	(0.0155)	(0.0154)	(0.0155)	(0.0154)
Female	-0.0894***	0.0507*	-0.0176	0.0298	-0.0924***
	(0.0313)	(0.0306)	(0.0305)	(0.0307)	(0.0304)
Education_middle	-0.0352	0.0156	-0.0356	0.0585	0.000385
	(0.0421)	(0.0411)	(0.0410)	(0.0412)	(0.0408)
Education_high	-0.117***	0.0628	-0.0377	0.130***	0.0148
	(0.0442)	(0.0432)	(0.0431)	(0.0434)	(0.0429)
Household_income	-0.0254*	-0.0101	-0.0134	-0.00274	-0.0192
	(0.0151)	(0.0147)	(0.0147)	(0.0148)	(0.0146)
Political_right	-0.0170	-0.00150	-0.0125	-0.0229	-0.00849
	(0.0163)	(0.0159)	(0.0159)	(0.0160)	(0.0158)
Voter	0.0464	0.0326	0.0458	-0.00701	-0.00104
	(0.0431)	(0.0421)	(0.0420)	(0.0423)	(0.0418)
Single_household	0.0921***	0.0638*	0.0759**	0.0447	0.0479
	(0.0345)	(0.0338)	(0.0336)	(0.0339)	(0.0335)
Urban	0.0126	0.00895	0.00702	0.00424	0.0251
	(0.0159)	(0.0155)	(0.0155)	(0.0156)	(0.0154)
Religious	-0.0461***	-0.00558	-0.00925	-0.0131	-0.0186
	(0.0163)	(0.0159)	(0.0158)	(0.0160)	(0.0158)
Constant	0.0801	-0.0345	0.0203	-0.0954*	0.0766
	(0.0564)	(0.0552)	(0.0550)	(0.0553)	(0.0547)
Observations	4,260	4,265	4,273	4,270	4,258
R-squared	0.025	0.067	0.068	0.065	0.085

(Standard errors in parentheses) *** p<0.01, ** p<0.05, * p<0.1

Note: The negative coefficients of the interaction *East_childhood*Born<1970* suggest that East-West differences in control aversion are larger for older Germans (born<1970) than for younger Germans (born >=1970). The size of the effect is substantial for most domains though imprecisely estimated.

Table S6. Linear OLS regressions on voluntary agreement. All dependent and independent variables are standardized (except for dummies). See table S3 for explanations of the independent variables.

	**Dependent variable: agreement if voluntary**				
	Use tracing app	Get vaccinated	Limit contacts	Limit travelling	Wear a mask
East_childhood	-0.105***	-0.0399	-0.149***	-0.0967**	-0.0626
	(0.0393)	(0.0402)	(0.0397)	(0.0408)	(0.0406)
Age	0.0214	0.0765***	0.0706***	0.0924***	0.101***
	(0.0214)	(0.0219)	(0.0216)	(0.0223)	(0.0221)
*East_childhood*Age*	0.0149	0.0197	-0.0396	-0.0706**	-0.00591
	(0.0337)	(0.0344)	(0.0341)	(0.0350)	(0.0349)
Trust_gov	0.186***	0.0587**	0.0591**	0.0101	0.0495*
	(0.0244)	(0.0250)	(0.0247)	(0.0254)	(0.0253)
Trust_science	0.0994***	0.0924***	0.147***	0.0996***	0.0822***
	(0.0205)	(0.0210)	(0.0207)	(0.0213)	(0.0212)
Trust_media	-0.00753	0.00706	-0.0484**	-0.0158	-0.0254
	(0.0196)	(0.0200)	(0.0198)	(0.0204)	(0.0202)
Cov19_truth_gov	0.0568**	0.0900***	0.131***	0.0661***	0.0610***
	(0.0221)	(0.0226)	(0.0224)	(0.0230)	(0.0229)
Trust_people	0.0133	0.00632	0.00848	0.0145	0.0361**
	(0.0160)	(0.0163)	(0.0162)	(0.0166)	(0.0165)
Cov19_risk_group	0.0490	0.0130	0.0502	0.0527	-0.0572*
	(0.0330)	(0.0337)	(0.0334)	(0.0343)	(0.0341)
Cov19_totalN	0.167*	0.194**	0.0346	-0.0748	0.136
	(0.0893)	(0.0914)	(0.0902)	(0.0928)	(0.0921)
Cov19_decline	-0.195**	-0.232**	-0.0770	0.0439	-0.176*
	(0.0887)	(0.0908)	(0.0896)	(0.0922)	(0.0915)
Date_time	-0.00776	0.0133	0.0265*	0.0310*	0.0177
	(0.0153)	(0.0156)	(0.0154)	(0.0159)	(0.0158)
Female	-0.0602**	-0.0534*	0.0409	0.0895***	0.00673
	(0.0302)	(0.0308)	(0.0305)	(0.0313)	(0.0311)
Education_middle	-0.0753*	-0.0548	-0.0181	0.0266	-0.0207
	(0.0403)	(0.0412)	(0.0408)	(0.0419)	(0.0417)
Education_high	-0.121***	0.0346	-0.00200	0.0858*	0.0164
	(0.0431)	(0.0441)	(0.0436)	(0.0448)	(0.0445)
Household_income	-0.0264*	-0.00336	-0.0355**	-0.0326**	-0.0386***
	(0.0144)	(0.0148)	(0.0146)	(0.0150)	(0.0149)
Political_right	0.0135	0.00786	0.0110	0.00211	0.00314
	(0.0156)	(0.0159)	(0.0157)	(0.0162)	(0.0161)
Voter	0.0465	0.0450	0.0505	-0.0282	-0.0173
	(0.0418)	(0.0428)	(0.0423)	(0.0435)	(0.0432)
Single_household	-0.0370	-0.00404	-0.0136	0.0174	-0.0156
	(0.0330)	(0.0338)	(0.0334)	(0.0344)	(0.0341)
Urban	0.0143	0.0182	0.00537	-0.00619	0.0206
	(0.0152)	(0.0155)	(0.0153)	(0.0158)	(0.0157)
Religious	-0.0146	-0.0457***	-0.0214	-0.0304*	-0.0168
	(0.0156)	(0.0159)	(0.0157)	(0.0162)	(0.0161)
Constant	0.0910*	0.00728	-0.0198	-0.0465	0.0702
	(0.0497)	(0.0509)	(0.0503)	(0.0517)	(0.0513)
Observations	4,275	4,276	4,277	4,272	4,265
R-squared	0.105	0.067	0.086	0.034	0.042

(Standard errors in parentheses) *** p<0.01, ** p<0.05, * p<0.1

Table S7. Linear OLS regressions on agreement under enforcement. All dependent and independent variables are standardized (except for dummies). See table S3 for explanations of the independent variables.

	**Dependent variable: agreement if enforced**				
	Use tracing app	Get vaccinated	Limit contacts	Limit travelling	Wear a mask
East_childhood	-0.0133	0.0769**	-0.00410	-0.0299	-0.0313
	(0.0375)	(0.0358)	(0.0359)	(0.0371)	(0.0358)
Age	0.0114	0.0899***	0.0292	0.0227	0.0399**
	(0.0205)	(0.0195)	(0.0195)	(0.0202)	(0.0195)
*East_childhood*Age*	0.0889***	0.0341	0.0147	0.00617	0.00949
	(0.0322)	(0.0308)	(0.0309)	(0.0318)	(0.0308)
Trust_gov	0.233***	0.162***	0.200***	0.170***	0.194***
	(0.0234)	(0.0223)	(0.0223)	(0.0231)	(0.0223)
Trust_science	0.0674***	0.146***	0.119***	0.122***	0.127***
	(0.0196)	(0.0187)	(0.0187)	(0.0193)	(0.0187)
Trust_media	0.0459**	0.0584***	0.0164	0.0158	0.0215
	(0.0187)	(0.0178)	(0.0179)	(0.0185)	(0.0179)
Cov19_truth_gov	0.130***	0.201***	0.251***	0.225***	0.230***
	(0.0212)	(0.0202)	(0.0202)	(0.0209)	(0.0202)
Trust_people	-0.0265*	-0.0350**	-0.112***	-0.0811***	-0.0605***
	(0.0153)	(0.0145)	(0.0146)	(0.0151)	(0.0146)
Cov19_risk_group	0.134***	0.125***	0.183***	0.163***	0.195***
	(0.0316)	(0.0300)	(0.0301)	(0.0311)	(0.0301)
Cov19_totalN	0.0399	0.148*	0.147*	0.0369	0.110
	(0.0853)	(0.0812)	(0.0815)	(0.0842)	(0.0813)
Cov19_decline	-0.0114	-0.146*	-0.112	-0.0169	-0.0708
	(0.0847)	(0.0807)	(0.0809)	(0.0836)	(0.0808)
Date_time	-0.0142	-0.0337**	-0.0263*	-0.0186	0.00914
	(0.0146)	(0.0139)	(0.0140)	(0.0144)	(0.0139)
Female	0.0399	-0.100***	0.0576**	0.0474*	0.122***
	(0.0288)	(0.0274)	(0.0275)	(0.0284)	(0.0275)
Education_middle	-0.0336	-0.0451	0.0309	-0.0448	-0.0263
	(0.0385)	(0.0367)	(0.0368)	(0.0380)	(0.0368)
Education_high	0.0105	-0.0133	0.0489	-0.0847**	-0.0126
	(0.0412)	(0.0393)	(0.0394)	(0.0407)	(0.0393)
Household_income	0.00241	0.00842	-0.0146	-0.0281**	-0.0117
	(0.0138)	(0.0131)	(0.0132)	(0.0136)	(0.0132)
Political_right	0.0316**	0.00706	0.0247*	0.0325**	0.0123
	(0.0149)	(0.0142)	(0.0142)	(0.0147)	(0.0142)
Voter	-0.00656	-0.0112	-0.0114	-0.0139	-0.00595
	(0.0400)	(0.0381)	(0.0382)	(0.0394)	(0.0381)
Single_household	-0.145***	-0.0821***	-0.102***	-0.0350	-0.0749**
	(0.0316)	(0.0301)	(0.0302)	(0.0312)	(0.0301)
Urban	0.000960	0.00335	-0.00357	-0.0136	-0.0125
	(0.0145)	(0.0138)	(0.0138)	(0.0143)	(0.0138)
Religious	0.0378**	-0.0353**	-0.00706	-0.0125	0.00819
	(0.0149)	(0.0142)	(0.0142)	(0.0147)	(0.0142)
Constant	-0.00270	0.0307	-0.0882*	-0.00786	-0.0853*
	(0.0475)	(0.0453)	(0.0454)	(0.0469)	(0.0453)
Observations	4,266	4,273	4,281	4,282	4,269
R-squared	0.189	0.261	0.257	0.207	0.258

(Standard errors in parentheses) *** p<0.01, ** p<0.05, * p<0.1

SI References

1. S. Rudorf, T. Baumgartner, S. Markett, K. Schmelz, R. Wiest, U. Fischbacher, D. Knoch, Intrinsic connectivity networks underlying individual differences in control-averse behavior. *Hum. Brain Mapp.* **39**, 4857-4869 (2018).
2. K. Schmelz, A. Ziegelmeyer, State Coercion and Control Aversion: Evidence from an Internet Study in East and West Germany. https://ideas.repec.org/p/twi/respas/0117.html (February 2020).
3. E. Fehr, B. Rockenbach, Detrimental effects of sanctions on human altruism. *Nature* **422**, 137-140 (2003).
4. S. Rudorf, K. Schmelz, T. Baumgartner, R. Wiest, U. Fischbacher, D. Knoch, Neural Mechanisms Underlying Individual Differences in Control-Averse Behavior. *The Journal of Neuroscience* **38**, 5196-5208 (2018).
5. S. Bowles, *The Moral Economy - Why Good Incentives are no Substitutes for Good Citizens* (Yale University Press, 2016).
6. Worldometer. *Worldometers coronavirus*, https://www.worldometers.info/coronavirus/country/germany/ (2020), accessed: May 30, 2020.
7. Y. Trope, N. Liberman, Construal-Level Theory of Psychological Distance. *Psychological Review* **117**, 440-463 (2010).
8. R. Hertwig, I. Erev, The description-experience gap in risky choice. *Trends Cogn. Sci.* **13**, 517-523 (2009).
9. R. Inglehart, C. Haerpfer, A. Moreno, C. Welzel, K. Kizilova, J. Diez-Medrano, M. Lagos, P. Norris, E. Ponarin & B. Puranen et al. (eds.). *World Values Survey: Round Six*, http://www.worldvaluessurvey.org/WVSDocumentationWV6.jsp (2014), accessed: June 15, 2020.
10. T. Fetzer, Witte, M., Hensel, L., Jachimowicz, J.M., Haushofer, J., Ivchenko, A., Caria, C., Reutskaja, E., Roth, C., Fiorin, F., Gomez, M., Kraft-Todd, G., Goetz, F., & Yoeli, E. *Global Behaviors and Perceptions in the COVID-19 Pandemic*, https://covid19-survey.org/ (2020), accessed: April 7, 2020.
11. Robert-Koch-Institut. *RKI Corona Bundesländer*, https://www.rki.de/DE/Content/InfAZ/N/Neuartiges_Coronavirus/Situationsberichte/Gesamt.html (2020), accessed: April to May, 2020.
12. J. F. Schulz, Kin Networks and Institutional Development. http://dx.doi.org/10.2139/ssrn.2877828 (27 April 2020).
